# The reliability of maternal audit instruments to assign cause of death in maternal deaths review process: a systematic review and meta-analysis

**DOI:** 10.1186/s12884-021-03840-3

**Published:** 2021-05-17

**Authors:** Ratnasari D. Cahyanti, Widyawati Widyawati, Mohammad Hakimi

**Affiliations:** 1 Doctoral Program, Faculty of Medicine, Public Health and Nursing, Universitas Gadjah Mada, Yogyakarta, Indonesia; 2 Obstetrics and Gynecology Department, Faculty of Medicine, Diponegoro University, Semarang, Indonesia; 3 Pediatric and Maternity Nursing Department, Faculty of Medicine, Public Health and Nursing, Universitas Gadjah Mada, Yogyakarta, Indonesia; 4 Obstetrics and Gynecology Department, Faculty of Medicine, Public Health and Nursing, Universitas Gadjah Mada, Yogyakarta, Indonesia

**Keywords:** Maternal death review, MDR instrument, Verbal autopsy, Facility-based MDR

## Abstract

**Background:**

Maternal Death Reviews (MDR) can assist in formulating prevention strategies to reduce maternal mortality. To support MDR, an adequate MDR instrument is required to accurately identify the underlying causes of maternal deaths. We conducted a systematic review and meta-analysis to determine the reliability of maternal death instruments for conducting the MDR process.

**Method:**

Three databases: PubMed, ProQuest and EBSCO were systematically searched to identify related research articles published between January 2004 and July 2019. The review and meta-analysis involved identification of measurement tools to conduct MDR in all or part of maternal audit. Eligibiliy and quality of studies were evaluated using the Modified Quality Appraisal of Diagnostic Reliability (QAREL) Checklist: Reliability Studies.

**Results:**

Overall, 242 articles were identified. Six articles examining the instrument used for MDR in 4 countries (4 articles on verbal autopsy (VA) and 2 articles on facility-based MDR) were included. None of studies identified reliability in evaluation instruments assessing maternal audit cycle as a comprehensive approach. The pooled kappa for the MDR instruments was 0.72 (95%CI:0.43–0.99; *p* < 0.001) with considerable heterogeneity (I^2^ = 96.19%; *p* < 0.001). Subgroup analysis of MDR instruments showed pooled kappa in VA of 0.89 (95%CI:0.52–1.25) and facility-based MDR of 0.48 (95%CI:0.15–0.82). Meta-regression analysis tended to show the high heterogeneity was likely associated with sample sizes, regions, and year of publications.

**Conclusions:**

The MDR instruments appear feasible. Variation of the instruments suggest the need for judicious selection of MDR instruments by considering the study population and assessment during the target periods.

**Supplementary Information:**

The online version contains supplementary material available at 10.1186/s12884-021-03840-3.

## Background

Despite the fact that the leading causes of maternal death are preventable, reducing maternal mortality rate (MMR) worldwide to become less than 70 per 100,000 live births by 2030, Sustainable Development Goals (SDGs) target 3.1, remains a challenge [1, 2]. Evidence has shown that the preventable maternal mortality events can be managed through proven health-care interventions including antenatal care, skilled care during delivery and care and support in the postnatal period [1, 3]. This evidence should be considered to support more proactive policy-making to prevent maternal deaths. To translate the evidence into action, maternal audits called Maternal Death Review (MDR) are required. The goal of the review is to develop a framework to understand the underlying factors contributing to maternal deaths, in order to stimulate and guide actions to prevent avoidable deaths [4, 5].

At the global level, preeclampsia and haemorrhage have been shown to be the leading direct causes of maternal deaths [6, 7]. At national and local levels, however, the contributing factors for these two direct causes of maternal deaths can be different. Accordingly, to provide the quality evidence for appropriate policy-making in these contexts, local MDR programs and initiatives are of primary importance [3, 8]. From several studies concerning MDR and other obstetrics audits conducted in Nigeria, synthesis findings reported that problems including delayed caesarean section, unavailability of magnesium sulphate and lack of safe blood transfusion service stand out as contributing factors of facility based-maternal deaths in the region. In contrast, research in Indonesia identified that poor implementation of standard operating procedures is the main problem found in root-cause analysis in the facility-based maternal deaths [9, 10].

To accomplish the elimination of this specific preventable event, the United Nation Commission on the Status of Women in 2012 proposed the important concept of maternal audit. The maternal audit is an action cycle that consists of defining cases, collecting data information, reviewing and analysing data, formulating and implementing recommendations, and re-evaluating current practices [4, 11]. The MDR serves as an essential component of the continuous action cycle of maternal audit. The reviews involve “qualitative, in-depth investigation of the leading causes and the avoidable factors of maternal death.” There are at least three common approaches to conduct MDR including verbal autopsies (at the community level), facility-based MDR (at the healthcare facility level), and the combination between the two approaches. The continuous action cycle of maternal audits must be repeated regularly for quality improvement. This action cycle is similar to that of the recent World Health Organization (WHO) Maternal Death Surveillance and Response (MDSR), in which the use of the term ‘response’ is intended to promote immediate action based on the findings. In the absence of appropriate responses, the maternal audit will be relatively meaningless and counterproductive [5, 11–14].

Despite the recommendations of the WHO, it is reported that most countries with high maternal mortality rates have no fully organized and structured MDR nor MDSR. The major obstacles identified related to the lack of implementation of maternal audits were stakeholder’s awareness, ‘blame culture’, lack of staff and training, poor data collection, poor follow-up and sustainability, and lack of financial resources. In addition, lack of knowledge and skills for conducting proper reviews can misclassify the causes of maternal deaths and lead to inappropriate recommendations and interventions. In fact, in developed countries with adequate civil registration systems, such misclassification has been shown to contribute to about 50% under-reporting of maternal deaths [5, 8, 12].

To accurately identify the underlying causes of maternal deaths, an adequate MDR instrument is required. The quality assessment of MDR can assist in the formulation of prevention strategies to reduce maternal mortality and morbidity. Several studies have indicated there are discrepancies in assigning the cause of death and determination of potential preventability between internal and external reviewers during the MDR process [5, 11]. Even in developed countries such as in the United States, research found that the external Maternal Mortality Review Committee (MMRC) identified 69.7% (*n* = 53) of cases potentially preventable, compared with 40.8% (*n* = 31) for the regional perinatal centers. The MMRC found more provider and systems factors compared with regional perinatal centers that claimed more patient factors for potentially preventable maternal deaths [15].

To reduce the potentially fatal results of these discrepancies, a valid reliable guideline to conduct MDR is highly in demand. Recently, no studies have rigorously evaluated the instrument properties in conducting standardized maternal audit reviews. To address this issue, we performed a systematic review and meta-analysis of existing literature to determine the reliability of MDR instruments in assessing of maternal causes of death.

## Methods

The performance reporting of the systematic review and meta-analysis followed the Preferred Reporting Items for Systematic Reviews and Meta-Analysis (PRISMA) statement [16]. The reliability of the instruments for conducting maternal death review was assessed [17]. Articles were systematically obtained from the three online databases: PubMed, ProQuest and EBSCO. The search was limited to English language articles. The article type was limited to journal articles and time of publication from January 2004 to July 2019. Backward citation tracking was performed to identify additional relevant articles. If the original publication lacked of adequate information details, electronic messages were sent to the corresponding authors. We did not attempt to extract unpublished data [18].

The selection of search terms was the following: (1) *construct of interest - maternal mortality or maternal death, review* or *audit* or *enquiry* or *inquiry* or *maternal death surveillance and response*; (2) target population – *maternal mortality*; (3) type of measurement instrument – (tool*) or (instrument*) and (4) measurement properties – reliable*, valid*, or *agreement.*

### Study eligibility criteria

The eligible articles were those reporting validity, reliability or agreement in conducting MDR. The excluded articles were non-English publications [19], and those assessing the general probable causes of death, quality improvement in maternal or perinatal services, or near-miss or severe maternal morbidity, or score of criterion-based clinical audit (CBCA), and studies with methodological problems.

### Study appraisal and synthesis methods

All titles and abstracts were independently screened by two reviewers (R.C., M.H). They scored the articles as “relevant” or “not relevant” based on the eligibility criteria. All of the potentially relevant full texts were independently assessed for eligibility by two review authors. The data from all relevant articles were extracted by both reviewers. Any disagreements or discrepancies between reviewers were resolved by discussion and if necessary by consultation to a third reviewer (W.W). There was 100% agreement in evaluation of the methodology quality between two authors. The following information was extracted from each study: author identification data, publication year, country and region of the study, sample size, study period, type of maternal death, electronic instruments, identify contributing condition of maternal death, validity of study, and standard coding of maternal death.

Next, the methodological quality of the studies was assessed using the Modified Quality Appraisal of Diagnostic Reliability (QAREL) Checklist: Reliability Studies [20, 21]. The instrument reliability was evaluated using kappa scores to estimate the inter-rater agreement to assign causes of maternal deaths. Kappa statistic (strength of agreement) was classified based on Landis and Koch criteria: poor (κ < 0.00), slight (κ = 0.00–0.20), fair (κ = 0.21–0.40), moderate (κ = 0.41–0.60), substantial (κ 0.61–0.80), and almost perfect (κ = 0.81–1.00) [22].

### Statistical analysis

The analysis held to get the value of pooled kappa which is the combined kappa value from the studies and random effect models were used to measure outcomes with heterogeneity. Risk of individual studies and assesment of publication bias were constructed through a visual inspection of forest and funnel plots. The degree of heterogeneity among the studies was assesed using the *I*^*2*^ statistic with *P* values. Values of 25, 50 and 75% were considered as low, moderate and high heterogeneity, respectively.

### Subgroup and meta-regression analysis

We conducted subgroup analysis based on type of maternal audit to appraise any potential source of heterogeneity. The meta-regression analysis was done to assess the potential sources of heterogeneity. Studies were stratified by sub-group of regions (Asia and Africa), sample-size (< 100 and ≥ 100) and year of publication (< 2011, 2011–2012, and > 2012). The statistical significance effect and their *p* values were calculated using random-effects meta-regression analysis.

## Results

**Fig. 1:**
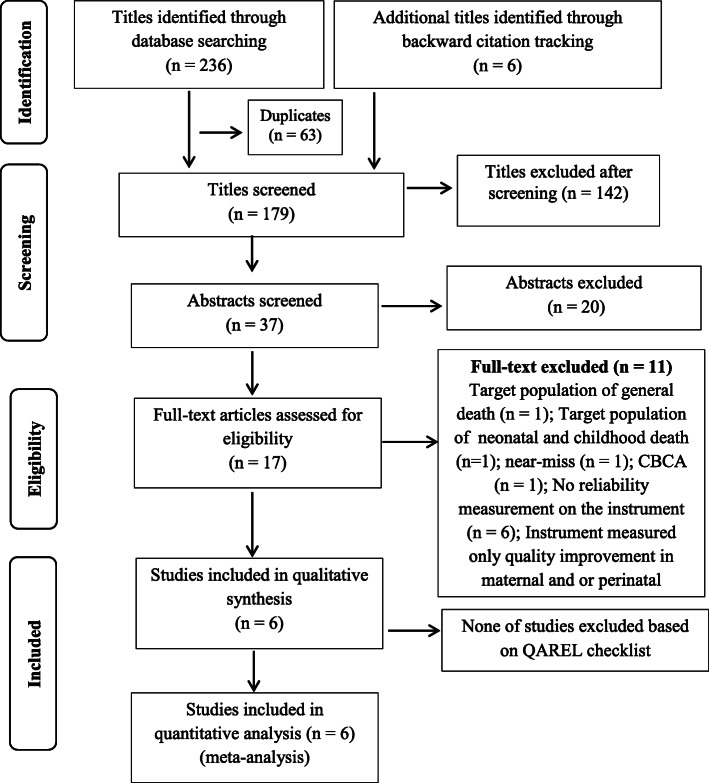
Study selection process

The selection procedure of articles is shown in Fig. 1. From the 242 identified articles, 17 full-text articles were subjected to eligibility assessment. Six eligible articles from 4 countries were included for the quality assessment [23–28].

As listed in Table 1, the included studies evaluated the reliability, construct, content, and criterion-related validity. Mostly, the studies provided the construct, content and criterion-related validity. Based on the classification of kappa score using the Landis and Koch criteria, the application of the the Maternal Data Extraction Tool (M-DET) in the study of VA in India conducted by Montgomery in 2011 was found to have the highest kappa score (κ = 0.85, 105 maternal deaths), however for their study conducted in 2012 with greater sample size, the value of kappa score was catagorized into substantial (κ = 0.66, 1040 maternal deaths) [25, 26].

**Table 1:** Systematic review of included articles

First Author, Year	Settings	Sample size	Type of maternal audit	Electronic instruments	Identify contributing conditions of maternal death	Validity				Standard of WHO coding of maternal death	κ
						Construct	Content	Criterion	Comparator Instrument		
Fottrell et al., 2007 [23]	Burkina Faso	258	Verbal autopsy	Yes InterVA-M	–	+	+	+	Origin physician review	–	κ = 0.80
Midhet, 2008 [24]	Pakistan	110	Verbal autopsy	No	–	–	+	–	Record assigned by doctor	–	κ = 0.378
Montgomery et al., 2011 [25]	India	105	Verbal autopsy	No M-DET (*Maternal Data Extraction Tool*)	(i) antenatal care accesss and final pregnancy outcome; (ii) planned place of birth and care provider; and (iii) consultation, transport, hospital admission, referral and number of health care contacts.	+	+	+	Trained physician vs midwife	ICD-10	κ = 0.85
Montgomery et al., 2012 [26]	India	1040	Verbal autopsy	No M-DET (*Maternal Data Extraction Tool*)	–	+	+	+	Independent trained physician review	ICD-10	κ = 0.66
Owolabi et al., 2014 [27]	Malawi	53	Facility-based MDR	No MDR International Classification of Disease Maternal Mortality (ICD-MM)	Clinical conditions	+	+	+	Healthcare provider using standard MDR form	ICD-MM	κ = 0.219
Mgawadere et al., 2016 (1) [28]	Malawi	86	Facility-based MDR	No MDR International Classification of Disease Maternal Mortality (ICD-MM)	Clinical conditions	+	+	+	Healthcare provider using standard MDR form	ICD-MM	κ = 0.37
Mgawadere et al., 2016 (2) [28]	Malawi	151	Facility-based MDR	No Expert panel using ICD-MM	Clinical conditions	+	+	+	InterVA-4 (electronic instrument VA)	ICD-MM	κ = 0.66

The study of reported M-DET was aimed to assign not only the underlying cause of maternal death but also to analysis the interrater-reliability of contributing conditions including: (i) antenatal care accesss and final pregnancy outcome; (ii) planned place of birth and care provider; and (iii) consultation, transport, hospital admission, referral and number of health care contacts. The agreement in the assignment of contributing conditions of maternal death in this M-DET VA was varied. They reported almost perfect agreement for determination of gestational age, pregnancy outcome, transport, death en route and admission to hospital as the contributing factors of maternal death. To determine of the cause of maternal mortality in the M-DET study, the ICD-10 cause of death was used to classify the obstetric cause of death [25].

ICD-MM, the only tool used for facility-based MDR in this review, focused on the contributory causes of maternal mortality related to clinical aspects. The ICD-MM was developed to solve the misinterpretation in determining the underlying (primary) and contributing (secondary) factors of maternal deaths [29]. The study of Owolabi in 2014 and Mgwadere in 2016 reported there was fair agreement between the facility-based review team and an expert panel using the ICD-MM classification (κ = 0.219, 53 maternal deaths and κ = 0.37, 86 maternal deaths, respectively). Because of the diverse variability of contributing factors, the levels of agreement among the groups cannot be accurately calculated [27, 28].

There were two studies which applied verbal autopsy (VA) using electronic instruments, known as InterVA-M and InterVA4 [23, 28]. In one study, the VA electronic instrument of InterVA4 was assessed as a comparator instrument to assign cause of maternal death using ICD-MM for healthcare providers in facility-based MDR. The agreement between the expert panel using ICD-MM and probabilistic model of INTERVA-4 was substantial (κ = 0.66, 151 maternal deaths). However, InterVA4 did not identify the contributing factors of maternal mortality [28].

The computer-probabilistic model (computer-coded VA) adapted from the general InterVA model to interprete VA data for deaths of women of reproductive age provided a consistent and reliable interpretation. The consistency of the InterVA-M model had a substantial kappa score (κ = 0.8, 258 maternal deaths) to assign a specific cause of maternal mortality [23].

Clearly established standards of the WHO coding of maternal deaths are required to classify the causes of maternal deaths. The articles on MDR conducted before 2010 used the physician review diagnosis as a main standard. This systematic review, in the context of M-DET, showed that the articles on MDR conducted after 2010 and before 2012 explicitly mentioned the use of ICD 10. Similarly, the study on MDR conducted in and after 2012 were shown to use the ICD-MM. None of the articles mentioned the utility of clinical standards to identify gaps and highlight deficiencies in the MDR process.

**Fig. 2:**
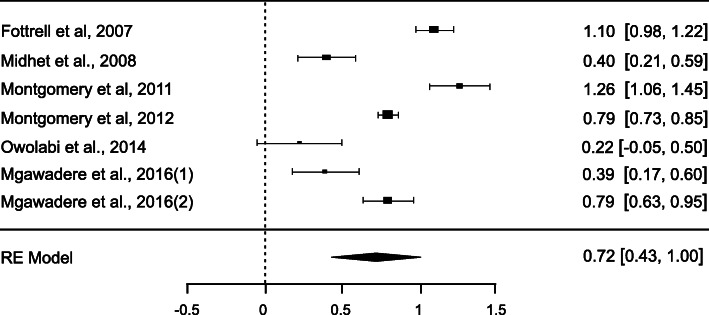
Forest plots of MDR instruments

**Fig. 3:**
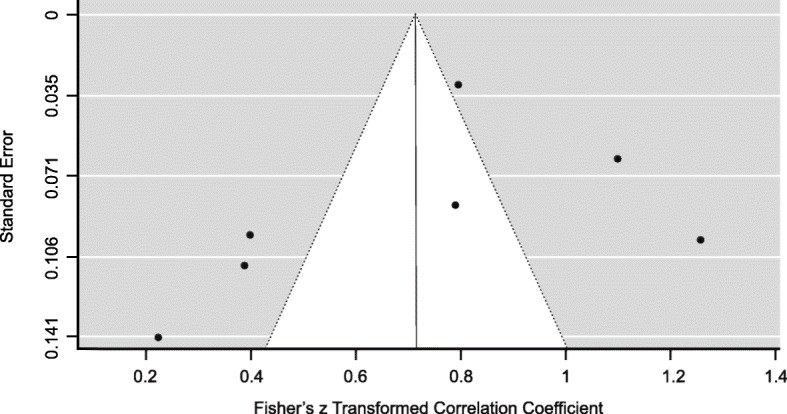
Funnel plots of publication bias

Table 2 represents a strong pooled of MDR instruments 0.72 (95% CI 0.43–0.99) in conducting this review. However, the agreement among the studies was considerably heterogeneous I^2^ = 96.19% (*p* < 0.001). The graphical funnel and forest plots (Figs. 2 and 3) were asymmetrical indicating a possible publication bias.

**Table 2:** Meta-analysis of maternal death review instruments

Meta-analysis	Pooled kappa	Random effect model			Heterogeneity test	
		95% CI		***p*** value	I^**2**^	***p***- value
		Lower	Upper			
MDR instruments	0.72	0.43	0.99	< 0.001	96.19%	< 0.001

The subgroup analysis based on the type of maternal audit (Table 3) highlighted the strong pooled kappa of VA 0.89 (95%CI: 0.52–1.25). Although VA and facility based-MDR had a correlation to assign cause of maternal death, there was a significant heterogeneity present in VA, I^2^ = 96.96% (*p* < 0.001) and facility based-MDR, I^2^ = 86.58% (*p* < 0.001).

**Table 3:** Sub-group analysis based on type of maternal audit

Type of maternal audit	Weight ratio	Pooled Kappa (95% CI)	***p***-value	Heterogeneity test	
				***p*** value	I^**2**^ (%)
**Verbal autopsy**		0.89 (0.52–1.25)	< 0.001	< 0.001	96.96
Fottrell et al., 2007 [23]	1.10				
Midhet et al., 2008 [24]	0.40				
Montgomery et al., 2011 [25]	1.26				
Montgomery et al., 2012 [26]	0.79				
**Facility-based MDR**		0.48 (0.14–0.82)	0.005	< 0.001	86.58
Owolabi et al., 2014 [27]	0.22				
Mgawadere et al., 2016 (1) [28]	0.39				
Mgawadere et al., 2016 (2) [28]	0.79				

The potential sources of heterogeneity (subgroups of regions, sample-size, and year of publications) were tested by meta-regression methods. There was significant association found among these variables (I^2^ > 92%, *p* value for heterogeneity < 0.001) (Table 4).

**Table 4:** Meta-regression analysis results

Type of maternal audit	Pooled Kappa (95% CI)	***p***-value	Heterogeneity test	
			***p***-value	I^**2**^ (%)
**Region**				
Asia	0.82 (0.34–1.29)	< 0.001	< 0.001	96.92
Africa	0.64 (0.25–1.03)	< 0.001	< 0.001	94.56
**Sample size**				
< 100	0.40 (0.11–0.45)	< 0.001	< 0.001	98.99
≥ 100	0.87 (0.59–1.15)	< 0.001	< 0.001	95.79
**Year of publication**				
< 2011	0.75 (0.51–1.21)	< 0.001	< 0.001	94.54
2011–2012	1.00 (0.57–1.25)	< 0.001	< 0.001	95.41
> 2012	0.58 (0.07–1.08)	0.025	< 0.001	95.24

## Discussion

Reliable identification of underlying causes of maternal deaths is an essential prerequisite to determine any existing barriers in preventing maternal mortality and formulate relevant targeted interventions [30, 31]. This systematic review and meta-analysis synthesized published evidence and quantified the reliability of maternal audit instruments in conducting MDR in community and facility-based settings. Our findings demonstrate that maternal audit instruments are potential tools to accurately determine the underlying cause of maternal deaths. Results of the subgroup analysis indicated that there was higher reliability of the MDR instruments in community settings than MDR in health facilities.

The cause for the discrepancies in the reliability of the studies has not been established. An assessment of the instrument development for MDR found terminology and regional variation leading to a possible discrepancy in evidence interpretation. The conceptual and contextual variations included: who conducted the review and when, where and how it was conducted. This present study supports several studies showing that inconsistencies in coding and assigning of the underlying cause of maternal deaths have been found across countries [23–28]. This may lead to difficulty for planning effective interventions intended to reduce maternal mortality and morbidity [27–29].

This present study aimed at determining the reliability of maternal death instruments for conducting the MDR. The VA studies showed a great variety of inter-rater reliability [23–28]. The highest inter-rater reliability in VA studies was found in the application of M-DET which determines not only the underlying cause but also the contributing factors of maternal mortality [25]. The identification of these contributing factors plays a significant role in the formulation of apropraite recommendations and actions related to the commonly accepted ‘three delays’ model of maternal deaths [31–33].

Clear criteria and guidance are required to accurately identify and classify the causes of maternal deaths due to the possible misinterpretation of available information. The main misclassification of maternal death occur in determining the symptoms, signs and diseases that must be reported and tabulated as the cause of mortality [27, 29]. The WHO provided a guideline to classify the causes of maternal deaths in 2010 called ICD-10 and its revised version published in 2012 is called the ICD-MM (International Statistical Classification of Diseases and Related Health Problems (ICD-10) for deaths in pregnancy, labor and puerperium). The version includes not only a guideline to classify maternal deaths but also guidelines to facilitate the consistent collection, analysis and interpretation of information on maternal mortality rates [29]. The articles in this study used the WHO classification of maternal death according to the classification in the year of study. The ICD-10 had become a standard of classification in the years of studies in 2011–2012 and the ICD-MM was applied for the studies after 2012 [25–28]. The standard classification of maternal death is one of the MDR end-points, but the application of universal standards to clarify and highlight the gaps and deficiencies of clinical management and health systems are the core results of quality improvement efforts. To reach the goals in strengthening the health system, the process analysis of MDR needs appropriate guidance for the key reviewers to identify the larger patterns of maternal deaths. In the absence of any clinical and health system standards to reflect the scientific evidence, the reviewers of MDR could be biased to reach a consensus on the appropriateness of the care provided [4, 34, 35].

The evidence stated that systematic methods to conduct a quality clinical review require clear criteria from evidence-based or clinical guidelines related to important aspects of health care, which should be measurable [36, 37]. Measurement of outcome can be used to identify problems in care, provided outcomes are clear, influenced by process, and occur within a short period of time. The criteria to assess the quality in healthcare and health systems can be categorized into structure (what you need); process (what you do) and outcome of care (what you expect). The benefit of categorizing the criteria is that if an outcome is not achieved and the necessary structure and processes have already been identified, then the main problems can be defined correctly [4, 36, 38].

### Policy implication

The study showed that to achieve the quality improvement of maternal health, a reliable MDR instrument is required. Besides the WHO standard on classification of maternal death, a standardized clinical management must be an integral part of MDR instrument to further identify the contributing factors of maternal death. This is particularly important because translating evidence from MDR will assist effective implementation of maternal health program based on local issues.

### Strength and limitation

This study highlighted the finding that even though notable efforts have been made to improve the quality of MDR by utilizing standardized guidelines to classify the causes of maternal deaths, but the evidence used as the reference standard and the techniques to identify the contributing events and clinical causes still were not identified. The specific limitations of this study should be considered: first, the number of studies included in the present meta-analysis is relatively small. Second, the heterogeneity of this study was statistically significant.

## Conclusions

In conclusion, our findings demonstrate that the available studies have variation of agreements in assessing the underlying causes of maternal deaths. Further meta-analysis studies should be done focusing on the specific region and type of maternal audit. The results suggest the need for a development of a universal MDR instrument to clarify and highlight the gaps and deficiencies of clinical management particularly for low- and middle-income countries.

## Supplementary Information

**Additional file 1.** Search strategies and results for the PubMed (accessed on 2019/08/01).

**Additional file 2.** Forest plot and funnel plot of the Maternal Death Review Instrument.

**Additional file 3: Table S1.** List of excluded studies along with reasons for exclusion.

## Data Availability

All data generated or analyzed during this study included in this published article and its additional files are available from the corresponding author upon reasonable request.

## Abbreviations

MDR: Maternal death review
QAREL: Quality appraisal of diagnostic reliability
SDGs: Sustainable development goals
VA: Verbal autopsy
MDSR: Maternal death surveillance and response
ICD-MM: ICD-10 to deaths during pregnancy, childbirth and puerperium
M-DET: Maternal Data Extraction Tool
