# Supplementary material for: The reliability of maternal audit instruments to assign cause of death in maternal deaths review process: a systematic review and meta-analysis
Source: BMC Pregnancy Childbirth. 2021 May 17;21:380. doi: 10.1186/s12884-021-03840-3 (PMC8127245; doi:10.1186/s12884-021-03840-3)
Supplement: Supplementary file 1 — Additional file 1. Search strategies and results for the PubMed (accessed on 2019/08/01). [file 12884_2021_3840_MOESM1_ESM.docx]

**Additional file 1** Search strategies and results for the PubMed (accessed on 2019/08/01)

|  | **Query** | **Result** |
| --- | --- | --- |
| #1 | Maternal Death Review [MeSH Terms] | 4540 |
| #2 | maternal death review [Title/Abstract] | 4327 |
| #3 | Maternal Audit [MeSH Terms] | 1372 |
| #4 | maternal audit [Title/Abstract] | 1258 |
| #5 | Maternal Death Enquiry [MeSH Terms] | 156 |
| #6 | maternal death enquiry [Title/Abstract] | 139 |
| #7 | Maternal Death Inquiry [MeSH Terms] | 10442 |
| #8 | maternal death inquiry [Title/Abstract] | 10254 |
| #9 | Maternal Death Surveillance and Response [MeSH Terms] | 308 |
| #10 | maternal death surveillance and response [Title/Abstract] | 302 |
| #11 | maternal death [Title/Abstract] | 21185 |
| #12 | Maternal Death [MeSH Terms] | 23885 |
| #13 | maternal mortality [Title/Abstract] | 34661 |
| #14 | Maternal Mortality [MeSH Terms] | 41098 |
| #15 | #1 OR #2 OR #3 OR #4 OR #5 OR #6 OR #7 OR #8 OR #9 OR #10 OR #11 OR #12 OR #13 OR #14 | 54033 |
| #16 | Tool [MeSH Terms] | 474333 |
| #17 | Instrument [MeSH Terms] | 120313 |
| #18 | Reliable [MeSH Terms] | 295652 |
| #19 | reliable [Title/Abstract] | 293252 |
| #20 | Reliability [MeSH Terms] | 162315 |
| #21 | reliability [Title/Abstract] | 157827 |
| #22 | Valid [MeSH Terms] | 111647 |
| #23 | validity [Title/Abstract] | 166058 |
| #24 | Agreement [MeSH Terms] | 268702 |
| #25 | agreement [Title/Abstract] | 266380 |
| #26 | #16 OR #17 OR #18 OR #19 OR #20 OR #21 OR #22 OR #23 OR #24 OR #25 | 1325406 |
| **#27** | **#15 AND #26** | **147** |

**Additional file 1** Search strategies and results for the EBSCO (accessed on 2019/08/05)

|  | **Query** | **Result** |
| --- | --- | --- |
| #1 | Maternal Death Review [MeSH Terms] | 183 |
| #2 | maternal death review [Title/Abstract] | 147 |
| #3 | Maternal Audit [MeSH Terms] | 98 |
| #4 | maternal audit [Title/Abstract] | 75 |
| #5 | Maternal Death Enquiry [MeSH Terms] | 181 |
| #6 | maternal death enquiry [Title/Abstract] | 125 |
| #7 | Maternal Death Inquiry [MeSH Terms] | 181 |
| #8 | maternal death inquiry [Title/Abstract] | 125 |
| #9 | Maternal Death Surveillance and Response [MeSH Terms] | 24 |
| #10 | maternal death surveillance and response [Title/Abstract] | 17 |
| #11 | maternal death [Title/Abstract] | 3205 |
| #12 | Maternal Death [MeSH Terms] | 3629 |
| #13 | maternal mortality [Title/Abstract] | 5825 |
| #14 | Maternal Mortality [MeSH Terms] | 10262 |
| #15 | #1 OR #2 OR #3 OR #4 OR #5 OR #6 OR #7 OR #8 OR #9 OR #10 OR #11 OR #12 OR #13 OR #14 | 11792 |
| #16 | Tool [MeSH Terms] | 337437 |
| #17 | Instrument [MeSH Terms] | 144520 |
| #18 | Reliable [MeSH Terms] | 58746 |
| #19 | reliable [Title/Abstract] | 56300 |
| #20 | Reliability [MeSH Terms] | 117406 |
| #21 | reliability [Title/Abstract] | 58924 |
| #22 | Valid [MeSH Terms] | 37021 |
| #23 | validity [Title/Abstract] | 65985 |
| #24 | Agreement [MeSH Terms] | 49940 |
| #25 | agreement [Title/Abstract] | 47049 |
| #26 | #16 OR #17 OR #18 OR #19 OR #20 OR #21 OR #22 OR #23 OR #24 OR #25 | 604073 |
| **#27** | **#15 AND #26** | **50** |

**Additional file 1** Search strategies and results for the ProQuest (accessed on 2019/08/07)

|  | **Query** | **Result** |
| --- | --- | --- |
| #1 | Maternal Death Review [MeSH Terms] | 98629 |
| #2 | maternal death review [Title/Abstract] | 956 |
| #3 | Maternal Audit [MeSH Terms] | 10484 |
| #4 | maternal audit [Title/Abstract] | 462 |
| #5 | Maternal Death Enquiry [MeSH Terms] | 14357 |
| #6 | maternal death enquiry [Title/Abstract] | 180 |
| #7 | Maternal Death Inquiry [MeSH Terms] | 14357 |
| #8 | maternal death inquiry [Title/Abstract] | 180 |
| #9 | Maternal Death Surveillance and Response [MeSH Terms] | 15848 |
| #10 | maternal death surveillance and response [Title/Abstract] | 34 |
| #11 | maternal death [Title/Abstract] | 7212 |
| #12 | Maternal Death [MeSH Terms] | 134754 |
| #13 | maternal mortality [Title/Abstract] | 10005 |
| #14 | Maternal Mortality [MeSH Terms] | 115191 |
| #15 | #1 OR #2 OR #3 OR #4 OR #5 OR #6 OR #7 OR #8 OR #9 OR #10 OR #11 OR #12 OR #13 OR #14 | 181316 |
| #16 | Tool [MeSH Terms] | 23453 |
| #17 | Instrument [MeSH Terms] | 11768 |
| #18 | Reliable [MeSH Terms] | 11865 |
| #19 | reliable [Title/Abstract] | 109313 |
| #20 | Reliability [MeSH Terms] | 8954 |
| #21 | reliability [Title/Abstract] | 81914 |
| #22 | Valid [MeSH Terms] | 9356 |
| #23 | validity [Title/Abstract] | 92238 |
| #24 | Agreement [MeSH Terms] | 14470 |
| #25 | agreement [Title/Abstract] | 137180 |
| #26 | #16 OR #17 OR #18 OR #19 OR #20 OR #21 OR #22 OR #23 OR #24 OR #25 | 397152 |
| **#27** | **#15 AND #26** | **39** |
