# Supplementary material for: The reliability of maternal audit instruments to assign cause of death in maternal deaths review process: a systematic review and meta-analysis
Source: BMC Pregnancy Childbirth. 2021 May 17;21:380. doi: 10.1186/s12884-021-03840-3 (PMC8127245; doi:10.1186/s12884-021-03840-3)
Supplement: Supplementary file 2 — Additional file 2. Forest plot and funnel plot of the Maternal Death Review Instrument. [file 12884_2021_3840_MOESM2_ESM.pdf]

Additional file 2 Forest plot and funnel plot of the Maternal Death Review Instrument

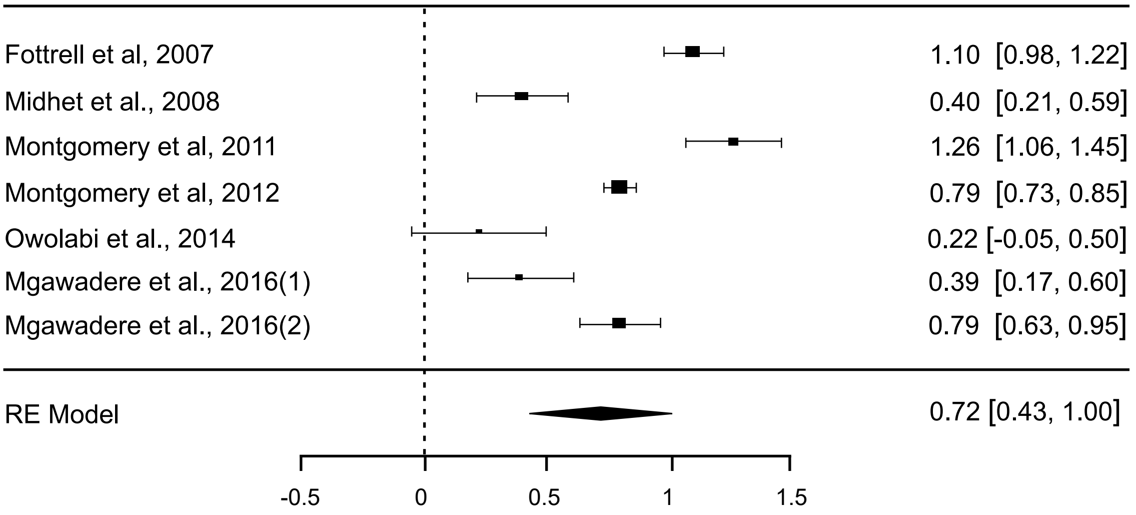

(a). Forest plots of MDR instruments

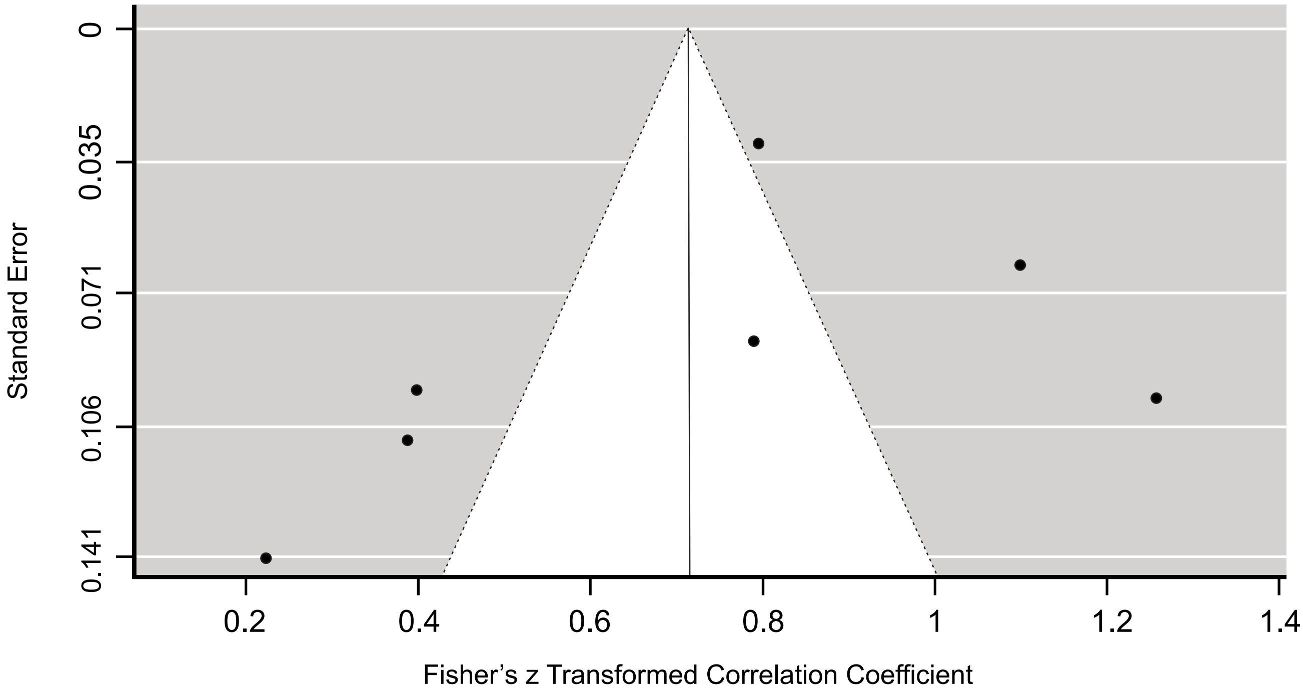

(b). Funnel plots of MDR instruments
