# Supplementary material for: The reliability of maternal audit instruments to assign cause of death in maternal deaths review process: a systematic review and meta-analysis
Source: BMC Pregnancy Childbirth. 2021 May 17;21:380. doi: 10.1186/s12884-021-03840-3 (PMC8127245; doi:10.1186/s12884-021-03840-3)
Supplement: Supplementary file 3 — Additional file 3: Table S1. List of excluded studies along with reasons for exclusion. [file 12884_2021_3840_MOESM3_ESM.docx]

**Table S1.** List of excluded studies along with reasons for exclusion

Full-text articles excluded, with reasons (n=11)

Target population of general death (n=1)

Target population of neonatal and childhood death (n=1)

Near-miss (n=1)

CBCA (n=1)

No reliability measurement on the instrument (n=6)

Instrument measured only quality improvement in maternal and or perinatal outcome (n=1)

| **Study** | **Reason for Exclusion** |
| --- | --- |
| Pirkle 2013 (1) | CBCA |
| Kalter 2011 (2) | No reliability measurement on the instrument |
| Kalter 2011 (3) | No reliability measurement on the instrument |
| Mitchell 2014 (4) | No reliability measurement on the instrument |
| Geller 2015 (5) | No reliability measurement on the instrument |
| Mpimbaza 2015 (6) | Target population of neonatal and childhood death |
| Hussain-Alkhateeb 2015 (7) | Target population of general death |
| Haddad (2012) (8) | Near-miss |
| Halder (2014) (9) | No reliability measurement on the instrument |
| Boyd (2017) (10) | No reliability measurement on the instrument |
| Esscher (2014) (11) | Instrument measured only quality improvement in maternal and or perinatal outcome |

**Reference:**

1. Pirkle CM, Dumont A, Traore M, Zunzunegui M-V. Validity and reliability of criterion based clinical audit to assess obstetrical quality of care in West Africa. BMC Pregnancy Childbirth. 2012;12(1):118.

2. Kalter HD, Salgado R, Babille M, Koffi AK, Black RE. Social autopsy for maternal and child deaths: A comprehensive literature review to examine the concept and the development of the method. Popul Health Metr. 2011;9:1–13.

3. Kalter HD, Mohan P, Mishra A, Gaonkar N, Biswas AB, Balakrishnan S, et al. Maternal death inquiry and response in India - the impact of contextual factors on defining an optimal model to help meet critical maternal health policy objectives. Heal Res Policy Syst. 2011;9:1–14.

4. Mitchell C, Lawton E, Morton C, McCain C, Holtby S, Main E. California pregnancy-associated mortality review: Mixed methods approach for improved case identification, cause of death analyses and translation of findings. Matern Child Health J. 2014;18(3):518–26.

5. Geller SE, Koch AR, Martin NJ, Prentice P, Rosenberg D. Comparing Two Review Processes for Determination of Preventability of Maternal Mortality in Illinois. Matern Child Health J. 2015;19(12):2621–6.

6. Mpimbaza A, Filler S, Katureebe A, Quick L, Chandramohan D, Staedke SG. Verbal autopsy: Evaluation of methods to certify causes of death in Uganda. PLoS One. 2015;10(6):1–14.

7. Hussain-Alkhateeb L, Fottrell E, Petzold M, Kahn K, Byass P. Local perceptions of causes of death in rural South Africa: A comparison of perceived and verbal autopsy causes of death. Glob Health Action. 2015;8(1):1–9.

8. Haddad SM, Sousa MH, Cecatti JG, Parpinelli MA, Costa ML, Souza JP. Intraclass correlation coefficients in the Brazilian network for surveillance of severe maternal morbidity study. BMC Pregnancy Childbirth. 2012;12(Icc).

9. Halder A, Jose R, Vijayselvi R. Maternal mortality and derivations from the WHO Near-Miss tool: An institutional experience over a decade in southern india. J Turkish Ger Gynecol Assoc. 2014;15(4):222–7.

10. Boyd AT, Hulland EN, Pierre RG, Nesi F, Honoré P, Jean-louis R. Use of Rapid Ascertainment Process for Institutional Deaths ( RAPID ) to identify pregnancy-related deaths in tertiary-care obstetric hospitals in three departments in Haiti. 2017;1–10.

11. Esscher A, Binder-Finnema P, Bødker B, Högberg U, Mulic-Lutvica A, Essén B. Suboptimal care and maternal mortality among foreign-born women in Sweden: Maternal death audit with application of the “migration three delays” model. BMC Pregnancy Childbirth. 2014;14(1):1–11.
